# RELATIONSHIP BETWEEN PARVOVIRUS B19 AND OSTEOARTHRITIS: LITERATURE REVIEW

**DOI:** 10.1590/1413-785220233103e267046

**Published:** 2023-07-17

**Authors:** GUILHERME LOTERIO MARQUES, THOMAS STRAVINSKAS DURIGON, FERNANDA CRISTINA DE SANTANA E SARTI, RENATO TADEU SASSMANNSHAUSEN MORETTO, FREDERICO CARLOS JANA NETO, GUILHERME GUADAGNINI FALÓTICO

**Affiliations:** 1 Universidade Nove de Julho, São Paulo, SP, Brazil.; 2 Universidade Federal de São Paulo, Escola Paulista de Medicina, São Paulo, SP, Brazil.; 3 Hospital Geral de Carapicuiba, Carapicuiba, SP, Brazil.; 4 Associação de Assistencia a Criança Deficiente, São Paulo, SP, Brazil.; 5 Instituto Prevent Senior, São Paulo, SP, Brazil.

**Keywords:** Osteoarthritis. Parvovirus B19, Human. Coinfeccion, Osteoartrite, Parvovírus B19 Humano, Coinfecção

## Abstract

Osteoarthritis and Parvovirus B19 infection present silent and gradual evolution, since the former is a degenerative process while the latter is often asymptomatic and may persist in the individual’s body during their life. This study aims to analyze clinical studies that establish a correlation between degenerative osteoarthritis and Parvovirus B19 infection. Of the 62 studies found, 25 were chosen for reading in full. Analyzing only the studies that establish the correlation between the pathologies, seven confirm this relationship between Parvovirus B19 and Osteoarthritis, while one reports no relationship. No objective correlation could be found between the other articles studied. Our findings suggest that there is a close relationship between Parvovirus B19 and Osteoarthritis, with a higher prevalence of acquired causes, women and older adults, but it can manifest during life. However, it is essential to carry out new studies involving family history of patients with Osteoarthritis with positivity of Parvovirus B19, cohort studies between childhood and adult-old adult, so that it can elucidate this duality of congenital-acquired cause and, finally, raise treatment alternatives. ***Level of Evidence II, Systematic Review of Level II Studies.***

## RESUMO

Tanto a osteoartrite quanto a infecção pelo parvovírus B19 apresentam evolução muitas vezes silenciosa e gradual, uma vez que a primeira é um processo degenerativo, e a segunda é geralmente assintomática, podendo persistir no corpo do indivíduo por toda a sua vida. Esta revisão bibliográfica visa analisar estudos clínicos que estabeleceram a correlação entre a osteoartrite degenerativa e a infecção pelo parvovírus B19. Dos 62 artigos encontrados, foram eleitos 25 para leitura em sua totalidade. Analisando apenas os artigos que estabelecem a relação entre as patologias, temos sete que confirmaram essa relação, enquanto um deles afirmou que não há relação. Não houve correlação objetiva entre os demais artigos estudados. Nossos resultados sugerem que há estreita relação entre a osteoartrite e o parvovírus B19, que tende a ser uma doença de causa adquirida com maior prevalência em mulheres e idosos, porém que pode se manifestar durante toda a vida. Contudo, é crucial a realização de novos estudos envolvendo antecedentes familiares de pacientes com osteoartrite e com positividade para o parvovírus B19 e estudos de coorte entre a infância e o adulto-idoso, a fim de elucidar essa dualidade de causa congênita-adquirida e, enfim, levantar alternativas de tratamento. **Nível de Evidência II, Revisão Sistemática de Estudos de Nível II.**

## INTRODUCTION

Human Parvovirus B19 is a single-stranded DNA virus, without membrane, and from the erythrovirus genus. This virus is responsible for a wide range of clinical manifestations still without specific antiviral therapy, only the treatment of symptoms.^1^ The target cells of parvovirus B19 are erythroid progenitors in the bone marrow, which induce changes in receptors, causing cell death by both lysis and apoptosis. ^(^ ^2^ The natural course of acute Parvovirus B19 infection is to be controlled by the immunocompetent individuals’ antibodies. ^(^ ^3^ Acute infection is characterized by skin rash, erythema infectiosum, arthralgia, fetal death, and transient aplastic crisis (TAC). However, in immunocompetent patients the pathogenesis is defined by viral symptoms: Fever, malaise, headache, nausea and sickness, and myalgia. Joint symptoms in Parvovirus B19 infection occur up to 80% in sick adults, most of them in women.

Osteoarthritis (OA) is a degenerative joint disease characterized by pain, especially during the morning, in at least one joint of the body, ^(^ ^4^ as well as stiffness, crepitation, and reduction of the joint space. ^(^ ^5^ Furthermore, OA is the most common disease of the locomotor apparatus and the major cause of disability, both in developed countries and in the so-called emerging countries. ^(^ ^6^ The traditional focus on treatment is given to the final stages of the disease with the placement of joint prosthesis, showing good effectiveness for the treatment of symptoms; however, low effectiveness in the treatment pathology itself. ^(^ ^7^ Currently, new treatments are emerging, aiming to replace the palliative treatment method with the preventive one, by gene-therapy and administration of intra-articular anti-inflammatory drugs seeking joint homeostasis. ^(^ ^8^

Thus, in this study, a medical literature search was carried out to identify the correlation between these pathologies, their pathophysiological processes, and currently used conducts.

This study aimed to analyze clinical studies that establish the correlation between Parvovirus B19 and osteoarthritis.

## METHODS

This is an integrative review with data collection performed from secondary sources, by a literature survey. A literature search of studies was performed using the following descriptors: Osteoarthritis, Osteoarthritis, Pavovirus B19 and Co-infection Pavovirus B19, and Osteoarthritis, in the following databases: Google Schoolar, PubMed, SciELO, Elsevier-ScienceDirect, and Web of Science. Inclusion criteria were analyzed studies with WebQualis A1 to C1. Exclusion criteria were studies related to other diseases besides those studied.

The studies were evaluated by their titles and abstracts. After applying the inclusion and exclusion criteria, they were read in full. The analysis of the selected studies and the synthesis of their extracted data were carried out, by at least two evaluators, in a descriptive way, enabling to observe, relate, and identify the evolution of the knowledge produced about the subject explored in this study.

## RESULTS

First, we found 62 studies, which were selected according to the inclusion and exclusion criteria. In total, 25 studies were eligible for reading in full. By analyzing only the studies that establish the relationship between the pathologies, we observed that seven confirmed the correlation between Parvovirus B19 and Osteoarthritis and, on the other hand, one of them reported no relationship. The other studies are about the pathologies studied and their concepts; however, without making an objective correlation.

Methods of analysis and research of the virus have become more sensitive and specific over the years, improving the samples accuracy. Among the positive studies found, other studies in the medical literature on the proposed topic are scarce, since there is no study establishing a direct relationship between Parvovirus B19 and autoimmune diseases, ^(^ ^9^ such as Osteoarthritis. The study by Rollín et al. ^(^ ^10^ stands out negatively regarding this correlation, since it reports that in patients with transplanted bone marrow the number of coinfected patients is insignificant to establish this relationship (Table 1).

**Table 1:** Analysis of studies on the correlation between parvovirus B19 and osteoarthritis.

Author	Date	Title	Correlation
Aslan et al. ^(^ ^11^	2008	Detection of parvovirus B19 in synovial fluids of patients with osteoarthritis.	Positive
Chen et al. ^(^ ^12^	2012	Significant association of past parvovirus B19 infection with cytopenia in both adult-onset Still's disease and systemic lupus erythematosus patients.	Positive
Colmegna and Alberts-Grill^13^	2009	Parvovirus B19: its role in chronic arthritis.	Positive
Kerr^14^	2000	Pathogenesis of human parvovirus B19 in rheumatic disease.	Positive
Meyer^15^	2003	Parvovirus B19 and autoimmune diseases.	Positive
Page et al. ^(^ ^9^	2015	Human parvovirus B19 and autoimmune diseases. Review of the literature and pathophysiological hypotheses	Positive
Rollín et al. ^(^ ^10^	2007	Human parvovirus B19, varicella zoster virus, and human herpesvirus-6 in mesenchymal stem cells of patients with osteoarthritis: analysis with quantitative real-time polymerase chain reaction.	Negative
Takahashi et al. ^(^ ^16^	1998	Human parvovirus B19 as a causative agent for rheumatoid arthritis.	Positive

## DISCUSSION

This study aimed to analyze the relationship of Parvovirus B19 in individuals with osteoarthritis (OA), focusing on the pathophysiological mechanism, epidemiology, and possible conducts. The results from most of the studies reviewed showed a relationship between both pathologies; however, they lacked evidence. This study showed that Parvovirus B19 has a greater tendency to be a disease of acquired cause, with virus positivity already in late adolescence^12^ and higher prevalence in older adults, especially women. ^(^ ^11^ ^),(^ ^13^ Rollín et al. ^(^ ^10^ ^)^ reinforce the acquired cause theory of the disease after obtaining a similar prevalence (16.7% and 20%) in their case-control study performed with young mesenchymal cell transplantation in patients with osteoarthritis, showing the absence of significant virus reactivation after transplantation. However, they do not specify that there is no relationship and that it cannot also be of congenital cause.

The prevalence of Parvovirus B19 in children is lower; however, it exists and has different clinical characteristics, ^(^ ^13^ which manifest themselves in an oligoarticular and asymmetrical way, preceded by erythema infectiosum (EI) and multiarticular in the adults and older adults, with a higher prevalence of the metacarpophalangeal, proximal interphalangeal, knee, wrist, and elbow joints, respectively. On the other hand, a case-control study, ^(^ ^11^ carried out to detect the virus in the synovial fluid in patients with osteoarthritis, reported no prevalence of involvement between the joints. There are no studies with any hypothesis as to why clinical characteristics between children and adults are different or not. Moreover, of the studies reviewed, we found no information whether people who tested positive for osteoarthritis have a previous history of PVB-19 infection; if the acquired cause is more prevalent from adolescence, ^(^ ^12^ or if in fact all these patients started the entire infectious trajectory in childhood, with the manifestations resulting from a recurrence or exacerbation of the virus. This shows the need for further evidence to corroborate the hypotheses in question.

One of the objectives of this study was to evaluate the impact of the presence of PVB-19 on joint damage. We observed that the persistence of the virus is crucial for the arthrogenic potential, although there is no conclusive evidence about its pathophysiological mechanism, according to Chen et al., ^(^ ^12^ Colmegna and Alberts-Grill, ^(^ ^13^ and Page et al. ^(^ ^9^ The viral proteins of Parvovirus B19, VP1 and NS1, trigger an inflammatory response that controls the expression of TNF-alpha and IL-6, leading to a cytotoxic action on the joints. Over time and with the persistence of inflammation, a set of events lead to increased joint destruction and clinical worsening, such as exacerbation of bone and cartilaginous loss, increased fibrosis, formation of osteophytes, among others. This corroborates the presence of PVB19 and IL-6 more frequently in individuals with stage 4 osteoarthritis, marked by visible deformities in the affected joint. ^(^ ^11^

Another important aspect of PVB19 is its correlation with autoimmune diseases. Among them, the most prevalent were Systemic Lupus Erythematosus, collagenosis, vasculitis, rheumatoid arthritis, among others. ^(^ ^9^ ^),(^ ^12^ ^),(^ ^14^ ^)-(^ ^16^ In an experimental study that showed apoptotic bodies (including autoantigens in SMITH-type autoimmunity, DNA, histone h4, and phosphatidylserine) generated by the expression of the protein PVB19-NS1 in an alternative, non-permissive cellular pathway. ^(^ ^9^ Another study conducted to associate PVB19 with rheumatic diseases found an increase in rheumatoid factor, antinuclear antibody, mitochondrial, and smooth muscle antibody in the presence of the virus. ^(^ ^14^ Only one study had a result without significance in the positivity between the IgG antibody of VP1 and NS1 and autoimmune diseases. However, authors still strongly believe in this relationship and that this topic needs further studies to draw a conclusion on the subject. ^(^ ^12^

Since PVB19 has an important role within autoimmune diseases and osteoarthritis is one of the common complications among this type of disease, there must be a close relationship between them three, so that intervention and one of the factors can reduce the effect of the others. The correct pathophysiological mechanism that induces the immune system to develop autoantibodies is unknown. Many hypotheses lead to other aspects of this discussion: one of them is that PVB19 has a terminal sequence similar to the adeno-associated adenovirus (AAV), which is not associated with human disease and can integrate and stabilize the human chromosome, ^(^ ^14^ thus making it more difficult to be recognized and eliminated by the immune system. Authors believe that there is a kind of “looping” between the parvovirus and the autoimmune diseases, which act as a trigger, one stimulating and exacerbating the other. ^(^ ^12^

This may explain why the persistence and recurrence of this type of virus is so frequent in individuals with autoimmune diseases that mainly involve joint disease. Furthermore, it also questions the clinical similarity between them, as well as the difficulty regarding a treatment; however, concrete evidence on these mechanisms is scarce. Takahashi et al. ^(^ ^16^ reported the clinical improvement of patients with rheumatoid arthritis and autoimmune hemolytic anemia associated with PVB-19 undergoing immunoglobulin therapy. However, many factors need to be interpreted: both the low sample of patients with rheumatoid arthritis examined and treated, ^(^ ^16^ and if immunoglobulin will be efficient considering the hypothesis previously discussed by Kerr, ^(^ ^14^ since the virus would stabilize on the human chromosome and may even act similarly to the autoantibody. If treatment would bring more benefits or harms is unknown. On the other hand, considering that this Parvovirus acts within a tripod between the virus-osteoarthritis-autoimmune disease, treating one of the causes may result in a decrease in the others. In the studies reviewed, we found no citations regarding other treatment alternatives besides immunoglobulin therapy; however, there is evidence of the use of anti-inflammatory drugs either intra-articularly, to improve isolated osteoarthritis not initially related to PVB-19, ^(^ ^8^ or systemically, if considering the hypothesis of persistence or exacerbation of the tripod Parvovirus-osteoarthritis-autoimmune disease.

## CONCLUSION

Our results suggest that there is a close relationship between Parvovirus B19 and osteoarthritis. However, the lack of a specific study for the correlation is highlighted, since it prevents a statistical deepening on the subject. We established an association that behaves predominantly as an acquired cause, with a higher prevalence among women and older adults, however, they can manifest during any age group. Although this study was unable to establish a specific causal relationship, there are plausible hypotheses that may justify the possible mechanisms of action and recurrence of the virus, which may act as a foundation for the discovery of new treatments. This subject should be further explored, with more studies involving family history of patients with osteoarthritis with positivity of Parvovirus B19, cohort studies between childhood and adult-old adults. Thus, it would be possible to elucidate this duality of congenital-acquired cause and, finally, raise treatment alternatives for this relationship.
